# Investigator experiences with financial conflicts of interest in clinical trials

**DOI:** 10.1186/1745-6215-12-9

**Published:** 2011-01-12

**Authors:** Paula A Rochon, Melanie Sekeres, John Hoey, Joel Lexchin, Lorraine E Ferris, David Moher, Wei Wu, Sunila R Kalkar, Marleen Van Laethem, Andrea Gruneir, Jennifer Gold, James Maskalyk, David L Streiner, Nathan Taback, An-Wen Chan

**Affiliations:** 1 Women's College Research Institute at Women's College Hospital, Toronto, Canada; 2 Department of Physiology University of Toronto, Toronto, Canada; 3 Dalla Lana School of Public Health, University of Toronto, Toronto, Canada; 4 School of Health Policy and Management, York University, Toronto, Canada; 5 Emergency Department, University Health Network, Toronto, Canada; 6 Department of Family and Community Medicine, University of Toronto, Toronto, Canada; 7 Clinical Epidemiology Methods Centre, Ottawa Health Research Institute; Department of Epidemiology and Community Medicine, Faculty of Medicine, University of Ottawa. Ottawa, Canada; 8 Toronto Rehabilitation Institute, Toronto, Canada; 9 Joint Centre for Bioethics, University of Toronto, Toronto, Canada; 10 Ontario Medical Association, Toronto, Canada; 11 Division of Emergency Medicine, University of Toronto, Toronto, Canada; 12 Department of Psychiatry, University of Toronto, Toronto, Canada

## Abstract

**Background:**

Financial conflicts of interest (fCOI) can introduce actions that bias clinical trial results and reduce their objectivity. We obtained information from investigators about adherence to practices that minimize the introduction of such bias in their clinical trials experience.

**Methods:**

Email survey of clinical trial investigators from Canadian sites to learn about adherence to practices that help maintain research independence across all stages of trial preparation, conduct, and dissemination. The main outcome was the proportion of investigators that reported full adherence to preferred trial practices for all of their trials conducted from 2001-2006, stratified by funding source.

**Results:**

844 investigators responded (76%) and 732 (66%) provided useful information. Full adherence to preferred clinical trial practices was highest for institutional review of signed contracts and budgets (82% and 75% of investigators respectively). Lower rates of full adherence were reported for the other two practices in the trial preparation stage (avoidance of confidentiality clauses, 12%; trial registration after 2005, 39%). Lower rates of full adherence were reported for 7 practices in the trial conduct (35% to 43%) and dissemination (53% to 64%) stages, particularly in industry funded trials. 269 investigators personally experienced (n = 85) or witnessed (n = 236) a fCOI; over 70% of these situations related to industry trials.

**Conclusion:**

Full adherence to practices designed to promote the objectivity of research varied across trial stages and was low overall, particularly for industry funded trials.

## Background

An estimated 20,000 trials are initiated internationally each year [1] with over 500 published each month [2]. Participants volunteer for these trials under the assumption that their efforts will contribute to the advancement of science. Accordingly, study results need to be objective, publicly available, and responsibly applied to advance knowledge and healthcare practice.

Concern about the potential impact of financial conflicts of interest (fCOI) on research conduct has led to recommendations for clinical trial practices designed to maintain an investigator's independence and to avoid the introduction of bias and suppression of results. Practices that promote the objectivity of research have been outlined in national standards for research ethics boards [3-6], requirements for federally funded research [3,7] guidance for academic institutions [8,9], requirements for trial registration [10] and for manuscripts submitted to biomedical journals [11-13]. We know little about adherence to these practices in the conduct of clinical trials.

We surveyed investigators about their experiences with practices designed to ensure the objectivity of research across all stages of industry and non-industry funded trials.

## Methods

### Survey Participants and Data Collection

We identified investigators conducting clinical trials at Canadian sites using the meta-register of Controlled Trials [14] to search for trials registered in *International Standard Randomized Controlled Trial Number *(ISRCTN) or *Clinicaltrials.gov*. E-mail addresses were obtained from the trial registry record or, if unavailable, from an internet search of public sources. Our study was approved by the research ethics board at the Baycrest Centre affiliated with the University of Toronto.

We identified 1,127 unique investigators based in Canada with a valid e-mail address. From May through November, 2006, we e-mailed potential respondents, asking them to complete an online questionnaire. A five dollar gift card was offered upon completion. Consent was considered to be implied when the investigators completed the on-line survey. Investigators were told that results will be presented as aggregate data only. Non-responders and those completing only a small portion of the questionnaire were sent up to five reminder e-mails at one-to two-week intervals. After excluding those who were unreachable (n = 18), defined as four auto-generated 'out of office' replies, the final sample was 1,109.

### Survey Design and Content

Our survey obtained information about an investigator's experience with situations related to fCOI that could introduce bias into a trial. The questionnaire content was based on the International Committee of Medical Journal Editors' (ICMJE) uniform requirements for manuscripts submitted to biomedical journals [11]; the fCOI literature; and the input of our research team (see Additional file 1). The ICMJE statement [11] was expanded in 2008 [12]. These criteria have been widely accepted by organizations including the World Association of Medical Editors (WAME) [15] and Consolidated Standards of Reporting Trials (CONSORT) [16]

Given that trials often take from four to eight years to conduct and publish, we asked investigators to describe their experiences over a five year time frame (2001-2006). We collected information about situations that could introduce bias during the stages of trial preparation (review of contracts and budgets, confidentiality clauses, trial registration), conduct (trial design, data access and ownership, data analysis and interpretation) and dissemination (manuscript preparation and publication). For each situation we identified what we considered to be preferred practice to protect an investigator's independence and to avoid the introduction of bias. Table 1 lists the preferred practices to promote the objectivity of research and the rationale for their inclusion.

**Table 1:** Survey questions related to preferred practices to promote the objectivity of research and their rationale

Practice	Rationale
Signed contracts reviewed by institution [18]	Reduce the risk of transparency bias

Signed contracts do not have restrictive confidentiality clauses that prevent disclosure of trial information without permission from the funder [17,18,29]	Reduce the risk of publication bias

Budgetary reviewed by a REB or institutional official [3]	Reduce the risk of transparency bias

Registration of a trial in a WHO approved registry since the requirement for trial registration in 2005 [10,30,31]	Reduce the risk of publication bias [32]

Investigators rather than funder should have data ownership [17,18,33]	Reduce the risk of reporting bias

Investigator should have access to data from all sites [11,12]	Reduce the risk of reporting bias

Funder should not control final decisions regarding	

Study design [11,12]	Reduce the risk of biased study designs

Data analysis [11,12]	Reduce the risk of biased analyses

Data interpretation [11,12]	Reduce the risk of biased interpretation

Funder should not control final decision on content of submitted manuscripts [11,12]	Reduce the risk of reporting bias

There should be no ghost authorship [34]	Reduce the risk of reporting bias

The survey questions related to preferred practices to promote the objectivity of research were based on the identified sources and the input of our research team.

#### Personal Experiences with fCOI

We asked investigators if they had ever experienced fCOI or witnessed a situation involving fCOI. If so, they were asked to describe the situation and whether these experiences took place in the context of an industry or non-industry funded trial.

### Statistical analysis

We used descriptive statistics to characterize respondents and their trial experience. Survey data were aggregated anonymously.

Our main study outcome was the percentage of investigators that reported full adherence to each preferred practice in *all *of their trials conducted from 2001-2006. Free text comments provided by investigators about their own personal or witnessed experiences with fCOI were summarized into thematic areas by two authors (SRK and WW). The initial agreement on the thematic areas was 91% for investigators' own experiences and 97% for experiences witnessed in a colleague. All differences were resolved by consensus.

## Results

Of 1,109 eligible investigators at Canadian sites, 844 (76%) responded. Among responders, 76 (7%) declined participation and 36 (3%) answered only the preliminary administrative questions. 732 investigators (included response rate, 66%) were in our final analysis. Of these, 32 did not provide information related to clinical trial experience but provided responses related to personal experiences with fCOI.

Almost all of the 732 investigators held primary university appointments. 67% had over five years of trial experience, and 64% had been the overall principal investigator for at least one trial (Table 2). More than 80% of investigators had participated in multi-site trials. Approximately half (n = 406) had been investigators on trials funded by both industry and non-industry sources.

**Table 2:** Investigator characteristics and clinical trial experience

Characteristics	Respondents (N = 732)
	n (%)
**Primary appointment**	
University or academic teaching hospital	684(93)
Non-academic community-based hospital	27(4)
Other (e.g. private practice, cancer centre, pharmaceutical)	21(3)
**Type of clinical trial**	
Non-industry trials only	240(33)
Industry trials only	54(7)
Both non-industry and industry trials	406(55)
None	28(4)
Did not answer	4(1)
**Number of years of experience in clinical trials**	
≤ 5	192(26)
> 5	489(67)
Not applicable	28(4)
Did not answer	23(3)
**Most senior role in clinical trial**	
Principal investigator for entire trial -(trial PI)	466(64)
Principal investigator for site, No overall PI experience- (site PI)	177(24)
Other (No PI or site-PI experience)	56(8)
Did not answer	33(5)
Intervention(s) studied *	
Drug therapy	552(75)
Device/equipment	217(30)
Diagnostic tests	174(24)
Surgery/procedure	151(21)
Education/counselling	139(19)
Management policy (e.g. specific thresholds for transfusion)	89(12)
Complementary and alternative medicine	78(11)
Psychotherapy	37(5)
Other (e.g. exercise, nutrition, radiation)	113(15)
**Trial sites**	
Single	94(13)
Multiple	252(34)
Both (single and multiple)	353(48)
Did not answer	33(5)
**Conflict of interest exposure**	
Any	269(37)
Personal only	33(5)
Witness of colleague	184(25)
Both personal and witness of colleague	52(7)
None	402(55)
Did not answer	61(8)

Note:

* The sum of response options is greater than 100% because survey respondents may have investigated more than one intervention type in different trials.

### 

#### Preferred Practices

700 investigators provided data about adherence to the practices designed to promote the objectivity of research in their non-industry (n = 646 investigators) and industry (n = 460) funded trials (Table 3).

**Table 3:** Adherence to the 11 preferred practices stratified by trial stage and funding

Practices	Trial Funding			
	
	Overall	Non-industry	Industry	Adherence to preferred practice (Non-industry vs. Industry)
	(N = 700)	(N = 646) *	(N = 460)^†^	
	n(%)	n(%)	n(%)	
***Trial Preparation Stage***				

**Signed contracts reviewed by institution ^‡^**				
Signed contracts	458	262	376	
No trials	13(3)	10(4)	10(3)	
Some trials	39(9)	21(8)	18(5)	
All trials ^§^	374(82)	191(73)	330(88)	Similar
Not sure	22(5)	32(12)	12(3)	
Did not answer	10(2)	8(3)	6(2)	

**Signed contracts have restrictive confidentiality clauses ^‡^**				
Signed contracts	458	262	376	
No trials ^§^	54(12)	48(18)	28(7)	Similar
Some trials	99(22)	35(13)	58(15)	
All trials	201(44)	77(29)	212(56)	
Not sure	94(21)	94(36)	72(19)	
Did not answer	10(2)	8(3)	6(2)	

**Budgetary reviewed by a research ethics board or institution official**				
No trials	39(6)	49(8)	22(5)	
Some trials	92(13)	56(9)	28(6)	
All trials ^§^	523(75)	487(75)	386(84)	Similar
Not sure	28(4)	38(6)	18(4)	
Did not answer	18(3)	16(2)	6(1)	

**Trials registered in trial registry since 2005**				
No trials	50(7)	56(9)	36(8)	
Some trials	221(32)	173(27)	69(15)	
All trials ^§^	274(39)	254(39)	141(31)	Similar
Not sure	140(20)	138(21)	193(42)	
Did not answer	15(2)	25(4)	21(5)	

***Trial Conduct Stage***				

**Funder owns study data**				
No trials ^§^	258(37)	394(61)	52(11)	Higher in non-industry
Some trials	221(32)	42(7)	114(25)	
All trials	107(15)	68(11)	172(37)	
Not sure	87(12)	119(18)	114(25)	
Did not answer	27(4)	23(3)	8(2)	

**Investigator has access to data from all sites**				
No trials	80(11)	69(11)	61(13)	
Some trials	191(27)	94(15)	108(23)	
All trials ^§^	265(38)	306(47)	99(22)	Higher in non-industry
Not sure	132(19)	147(23)	181(39)	
Did not answer	32(5)	30(5)	11(2)	

**Funder controls final decisions regarding**:				
**Study design**				
No trials ^§^	247(35)	366(57)	78(17)	Higher in non-industry
Some trials	228(33)	46(7)	112(24)	
All trials	141(20)	118(18)	179(39)	
Not sure	63(9)	98(15)	84(18)	
Did not answer	21(3)	18(3)	7(2)	

**Data analysis**				
No trials ^§^	276(39)	397(61)	92(20)	Higher in non-industry
Some trials	222(32)	37(6)	114(25)	
All trials	120(17)	109(17)	155(34)	
Not sure	61(9)	85(13)	92(20)	
Did not answer	21(3)	18(3)	7(2)	

**Data interpretation**				
No trials ^§^	300(43)	404(63)	103(22)	Higher in non-industry
Some trials	207(30)	36(6)	111(24)	
All trials	106(15)	106(16)	126(27)	
Not sure	66(9)	82(13)	113(25)	
Did not answer	21(3)	18(3)	7(2)	

***Trial Dissemination Stage***				

**Funder controls final decision on content of submitted manuscripts**				
No trials ^§^	368(53)	445(69)	124(27)	Higher in non-industry
Some trials	168(24)	37(6)	100(22)	
All trials	49(7)	41(6)	70(15)	
Not sure	88(13)	100(15)	157(34)	
Did not answer	27(4)	23(4)	9(2)	

**Completed manuscripts has ghost authorship**				
No trials ^§^	450(64)	478(74)	147(32)	Higher in non-industry
Some trials	100(14)	35(5)	75(16)	
All trials	5(1)	4(1)	8(2)	
Not sure	117(17)	104(16)	220(48)	
Did not answer	28(4)	25(4)	10(2)	

Notes:

* 646 investigators included 406 who had experience in *both *industry funding and non-industry funding trials and 240 who only had experience in non-industry funding trials. We defined non-industry funding as support from a government agency, hospital, university, or other non-profit source (e.g., a federal granting organization) and industry funding as support from a private for-profit corporation (e.g., pharmaceutical company).

^† ^460 investigators included 406 who had experience in *both *industry funding and non-industry funding trials and 54 who only had experience in industry funding trials.

^‡ ^Question was related to 458 investigators who had signed contracts.

^§ ^Rows indicated the proportion of investigators that reported full adherence to the specific preferred trial practice in *all *of their trials experience

Overall, in the trial preparation stage, 458 (65%) investigators had a signed contract for one or more trials. Of these, 374 (82%) investigators reported always having the contracts reviewed by the research ethics board (REB) or institution and 54 (12%) reported no restrictive confidentiality clauses within the contract. 523 (75%) reported always having their budgets reviewed by their REB or institution, and 274 (39%) reported always having their trials registered (since 2005). For these 4 practices, full adherence was similar between industry and non-industry trials.

In the trial conduct stage, less than half of investigators reported full adherence to preferred practices in all of their trials with regards to data ownership (37%); data access (38%); control over study design (35%); data analysis (39%); and data interpretation (43%). In the trial dissemination stage, 368 (53%) investigators reported always having ultimate control over the contents of submitted manuscripts and 450 (64%) reported an absence of ghost authorship in all their manuscripts. In addition to these reports of full adherence to preferred practice, other investigators reported following these preferred practices in some trials, but not all trials. Full adherence to preferred practices in the trial conduct and dissemination stages was generally higher for non-industry relative to industry funded trials.

We also stratified investigators according to whether they had experience in only a single funding environment or in both industry and non-industry funding environments and compared the frequency of preferred practices between industry and non-industry funded trials within these strata. We found no differences in the overall pattern of responses in either stratum. These results are not reported here but are available in Additional file 2.

#### Personal Experience with fCOI

Overall, 269 (37%) investigators reported having personally experienced or witnessed a situation involving fCOI (Table 4). These experiences were personal (n = 33), witnessed in a colleague (n = 184), or both (n = 52). Of 85 investigators who personally experienced a fCOI situation, the most frequent theme was related to recruitment (33%). Another theme involved study conduct (24%). 61 (72%) indicated that these fCOI experiences involved industry-funded trials.

**Table 4:** Thematic description of personal conflicts of interest situations

**Financial Conflict of Interest**	**Experienced by Investigators**		**Witnessed in Colleagues Research**	
		
	**Number (%)** **(N = 85)**	**Examples from Free Text Comments**	**Number (%)** **(N = 236)**	**Examples from Free Text Comments**
Recruitment	28 (33)	• *Pressure to recruit patients*	31 (13)	• *Investigators receive direct benefit from enrolling patients*
Study conduct	20 (24)	• *Being asked to have a paper ghost-written*	37 (16)	• *Review focusing on trials with positive result*
Personal financial incentives	9 (11)	• *Possible financial gain by success of a drug/study*	56 (24)	• *Equity holding in a company whose product is being investigated*
Conflicting roles	6 (7)	• *Invented and patented devices and involved in their study*	19 (8)	• *Being the site principal investigator and the patient's physician*

Of 236 investigators who reported witnessing a fCOI situation in a colleague's research, the most frequent theme related to personal financial incentives (24%) (Table 4). 180 (76%) of respondents indicated that the situations they witnessed were in relation to industry-funded trials.

## Discussion

To our knowledge, our study is the first to obtain information directly from investigators about practices related to fCOI that may introduce bias into a trial at the preparation, conduct, and dissemination stages. Previous studies have largely relied on information obtained from indirect sources. For example, information on restrictive confidentiality clauses has come from surveys of medical school research administrators [17] while information on investigator participation in trial design, data access, and publications has come from surveys of medical schools officials [18]. Court documents have been the source of information for much of what we know about the practice of ghost authorship [19-23]

Our findings suggest that full adherence to preferred practice was highest when these practices are required and enforced by an external agent. Specifically, three quarters of investigators reported that all of their contracts and budgets were reviewed by an REB or an institutional official. Further, these practices were equally likely to occur in industry and non-industry funded trials. The high rate of compliance may reflect the requirement of institutions to review contracts and vigilance that ethics board members apply when they review studies [24]. Adherence to trial registration was also similar for industry and non-industry trials after 2005 (when registration became a precondition for publication in an ICMJE journal [13]). Registration has been a legal requirement for all trials of interventions receiving regulatory approval in the United States since 2007 [10] and has been included in the World Medical Association Declaration of Helsinki since 2008 [25].

We found that adherence was lowest for preferred practices outlined by ICMJE regarding trial conduct and dissemination. There are a number of possible explanations for this result. First, these practices are recommended but not required by all medical journals. Second, the ICMJE recommendations generally target disclosure of information at the publication stage of the trial. Guidance introduced earlier in the process would alert investigators to preferred practices and encourage their incorporation into the study design. A fCOI Checklist [26] aimed at prospectively identifying investigator fCOI in trials has been recently developed. To facilitate the conduct of preferred practices throughout the course of a clinical trial, this fCOI Checklist is intended to be initiated during the trial preparation stage and continues through to the trial's result dissemination stage [26].

Our data are consistent with previous evidence that a substantial proportion of trials have ghost authorship [19,21,22]. Less than a third of surveyed individuals indicated that ghost authorship was absent in all of their industry sponsored trials experience compared to more than two thirds for non-industry trials. A coordinated oversight strategy has been proposed to address this problem [19]. Increased awareness of this issue is important so that investigators understand the potential bias introduced by ghost authors.

Our findings are robust given that our original survey was worded so that investigators responded without explicit knowledge of the preferred behaviour. Additionally, identical questions were used to capture industry and non-industry funded trial experience. Our large number of respondents and reasonable response rate indicates the willingness of investigators to discuss potentially sensitive issues concerning their experiences. Our findings also describe the experiences of individual investigators. More than a third reported having personally experienced or witnessed a situation of potential fCOI, mostly in industry-funded trials. One of the most frequently described situations related to recruitment pressures. Our study indicates the need to explore this issue further.

### Limitations

First, our sample of Canadian investigators may not reflect the perspectives of investigators globally. Increasingly, clinical research sites are moving to areas such as Eastern Europe and Latin America that may have less experience with clinical trials [27]. Second, our sample included only registered clinical trials. Since, registration has been a precondition for publication in an ICMJE journal since 2005 [13] the trials included in our sample may have been of higher quality than trials that were not registered. Some of the trials included in our sample pre-date the mandatory registration period. Third, response bias is a concern, particularly when addressing potentially sensitive issues involving fCOI. Our guarantee of anonymity, and user-friendly questions helped to encourage disclosure of useful information. The response rate to our email survey was 76% with 66% useable responses. We have no information from non-responders and therefore are unable to describe these individuals. Further, our main study outcome was full adherence to preferred practice in all of their trials experiences within 5-years of our survey. We recognize that other surveyed investigators followed the preferred practices in some but not all trials. Finally, we surveyed investigators about their trial experience prior to 2007. Since we aimed to capture practices across all stages of clinical trial conduct and study result dissemination (average 4 to 8 years from inception to completion [28]), we needed to allow sufficient time for publication. Our results may not fully reflect current practices but they provide a baseline from which future studies can build.

## Conclusions

Full adherence to practices designed to promote the objectivity of research varied across trial stages and was low overall, particularly for industry funded trials. Adherence to preferred practices was highest when they were required by an external agent. Guidance introduced early in the trial process could alert investigators to preferred practices and encourage their incorporation into the study design.

## Competing interests

Joel Lexchin was retained by a law firm representing Apotex to provide expert testimony about the effects of promotion on the sales of medications. He has also been retained as an expert witness by the Canadian federal government in its defense of a law suit launched challenging the ban on direct-to-consumer advertising of prescription drugs in Canada. No conflicts reported for rest of the authors.

## Funding Support

This project was funded by Canadian Institutes of Health Research (CIHR) Grant entitled Evaluation of the Integrity of Clinical Research in Canada EIC - 77338. The funding organizations did not participate in the design or conduct of the study, in the collection, analysis, or interpretation of the data, or in the preparation, review, or approval of the manuscript. Dr. Rochon had full access to all of the data in the study and takes responsibility for the integrity of the data and the accuracy of the data analysis.

## Authors' contributions

PAR, JH, JL, LEF, DM, MVL, JG, JM, DS, NT, AWC conceived the project; PAR, JH, JL, LEF, DM, JG, JM, DS, NT, AWC obtained the funding; PAR, MS, JH, JL, LEF, DM, MVL, JG, JM, NT, AWC participated in the design of the survey; MS, SRK were research assistants on the study and were involved in data collection; PAR, MS, JH, JL, LEF, DM, WW, SRK, MVL, AG, JM, DLS, NT, AWC participated in analyzing the research; PAR, MS, JH, JL, LEF, DM, WW, SRK, MVL, AG, JG, JM, DLS, NT, AWC helped to draft the manuscript, and approved the final manuscript

PAR is the study guarantor.

## Supplementary Material

Additional file 1 **Survey questions**. Click here for file

Additional file 2 **Adherence to the 11 preferred practices stratified by trial stage and funding**. Click here for file
