# Supplementary material for: Investigator experiences with financial conflicts of interest in clinical trials
Source: Trials. 2011 Jan 12;12:9. doi: 10.1186/1745-6215-12-9 (PMC3031202; doi:10.1186/1745-6215-12-9)
Supplement: Additional file 1 — Survey questions. [file 1745-6215-12-9-S1.DOC]

**Additional file 1: Survey questions**

1. Is your primary appointment: (University or Academic Hospital; Non-academic community-based hospital; Other)

**Question 2 -21 were related to investigators’ experience in non-industry funded trials:**

2. Have you participated in a non-industry funded clinical trial as an investigator since the beginning of 2001? (Yes/No)

3. In what calendar year did you participate in your first non-industry funded clinical trial as an investigator? (Select a year)

4. In approximately how many non-industry funded clinical trials have you participated as an investigator since the beginning of 2001? (1-4; 5-10; 11-15; +15)

5. What has been your role in non-industry funded clinical trials since the beginning of 2001? (Principal investigator for the entire trial; Principal investigator for your site or region only; Member of coordinating committee; Investigator who recruited participants; Investigator who designed the trial; Investigator who collected data; Investigator who analyzed data; Investigator who interpreted final trial results; Other)

6. Did these non-industry funded clinical trials involve data collection from single or multiple study sites? (Single site; Multi-site; Both single- and multi-site)

7. What was (were) the intervention(s) under investigation in these trials? (Complementary and alternative medicine; Device/Equipment; Diagnostic Tests; Drug; Education/Counseling; Management Policy; Psychotherapy; Surgery/procedure; Other)

8. Among non-industry-funded trials in which you have participated since the beginning of 2005, what percent have been registered in a clinical trial register such as that mandated by the World Health Organization and the International Committee of Medical Journal Editors (ICMJE)?

9. For what percent of non-industry funded trials were you offered a contract by the funding source?

10. What percent of contracts that you were offered included a confidentiality clause? A confidentiality clause is defined here as an agreement not to disclose any or all information about a trial without permission from the funding source.

11. If you were offered a contract, for what percent of non-industry funded trials did you sign the contract you were offered?

12. If contracts for these non-industry funded trials were signed, what percent had their content reviewed by your institution?

13. What percent of signed contracts had confidentiality clauses?

14. What percent of non-industry funded trials had their budgets reviewed by the research ethics board or by an official of the institution?

According to the trial protocol or contract, for what percent of non-industry funded trials did the funder maintain the right to control the final decision about each of the following?

15. Study Design

16. Data Analysis

17. Data Interpretation

18. In what percent of non-industry funded trials did the funding source own the study data?

19. In what percent of non-industry funded trials did one or more investigators have access to all data from all sites?

20. According to the protocol or contract, for what percent of non-industry funded trials did the funding source reserve the right to control the final decision about the content of manuscripts submitted for publication?

21. For what percent of non-industry funded trials with completed manuscripts was ghost authorship present?

**Question 22 -41 were related to investigators’ experience in industry funded trials:**

22. Have you participated in an industry-funded clinical trial as an investigator since the beginning of 2001? (Yes/No)

23. In what calendar year did you participate in your first industry-funded clinical trial as an investigator? (Select a year)

24. In approximately how many industry funded clinical trials have you participated as an investigator since the beginning of 2001? (1-4; 5-10; 11-15; +15)

25. What has been your role in industry funded clinical trials since the beginning of 2001? (Principal investigator for the entire trial; Principal investigator for your site or region only; Member of coordinating committee; Investigator who recruited participants; Investigator who designed the trial; Investigator who collected data; Investigator who analyzed data; Investigator who interpreted final trial results; Other)

26. Did these industry-funded clinical trials involve data collection from single or multiple study sites? (Single site; Multi-site; Both single- and multi-site)

27. What was (were) the intervention(s) under investigation in these trials? (Complementary and alternative medicine; Device/Equipment; Diagnostic Tests; Drug; Education/Counseling; Management Policy; Psychotherapy; Surgery/procedure; Other)

28. Among industry funded trials in which you have participated since the beginning of 2005, what percent have been registered in a clinical trial register such as that mandated by the World Health Organization and the International Committee of Medical Journal Editors (ICMJE)?

29. For what percent of industry-funded trials were you offered a contract by the funding source?

30. What percent of contracts that you were offered included a confidentiality clause? A confidentiality clause is defined here as an agreement not to disclose any or all information about a trial without permission from the funding source.

31. If you were offered a contract, for what percent of industry funded trials did you sign the contract you were offered?

32. If contracts for these industry-funded trials were signed, what percent had their content reviewed by your institution?

33. What percent of signed contracts had confidentiality clauses?

34. What percent of industry funded trials had their budgets reviewed by the research ethics board or by an official of the institution?

According to the trial protocol or contract, for what percent of industry-funded trials did the funder maintain the right to control the final decision about each of the following?

35. Study Design

36. Data Analysis

37. Data Interpretation

38. In what percent of industry-funded trials did the funding source own the study data?

39. In what percent of industry-funded trials did one or more investigators have access to all data from all sites?

40. According to the protocol or contract, for what percent of industry-funded trials did the funding source reserve the right to control the final decision about the content of manuscripts submitted for publication?

41. For what percent of industry funded trials with completed manuscripts was ghost authorship present?

42. In approximately how many partial industry-funded clinical trials have you participated as an investigator since the beginning of 2001? (0; 1-4; 5-10; 11-15; +15)

43. Are you aware of any policies or guidelines related to conflict of interest in clinical research at the institution where you hold your primary appointment? (Yes/No)

44. Do you know how to access policies/guidelines pertaining to conflicts of interest? (Yes/No)

45. In your opinion, have you ever experienced a conflict of interest that could potentially compromise the integrity of your research? (Yes/No)

46. If you choose to, please describe the nature of this conflict of interest in a few sentences: (Open-Ended Response)

47. Was/were the influenced study/studies funded by: (Industry/Non-industry)

48. If you choose to, please describe in a few sentences how you resolved the conflict: (Open-Ended Response)

49. Did you consult a conflict of interest or related policy or consult someone familiar with conflict of interest policies/guidelines at your institution? (Yes/No)

50. Did you find the conflict of interest policy/guideline at your institution to be helpful? (Yes/No)

51. Have you ever been aware of a conflict of interest that could potentially compromise the integrity of a colleague's research? (Yes/No)

52. If you choose to, please describe the nature of your colleague's conflict in a few sentences: (Open-Ended Response)

53. If you were aware of a conflict of interest that could potentially compromise the integrity of a colleague's research, was the funding source: (Industry/Non-industry)

54. Which guidelines related to conflict of interest, research ethics, or scientific integrity do you consult when participating as an investigator? (The Belmont Report; Conflicts of interest policies at your institution; Declaration of Helsinki; International Guidelines for Ethical Review of Epidemiological Studies; ICH Guidelines for Good Clinical Practice; Nuremberg Code; Tri-Council Policy Statement: Ethical Conduct for Research Involving Humans; Tri-Council Policy Statement: Integrity in Research and Scholarship; Uniform Requirements for Manuscripts Submitted to Biomedical Journals: Writing and Editing for Biomedical Publication; None; Other)

55. If you choose to, please describe briefly what you, as an investigator, would find useful in a conflict of interest policy/guideline statement: (Open-Ended Response)
