# Supplementary material for: Investigator experiences with financial conflicts of interest in clinical trials
Source: Trials. 2011 Jan 12;12:9. doi: 10.1186/1745-6215-12-9 (PMC3031202; doi:10.1186/1745-6215-12-9)
Supplement: Additional file 2 — Adherence to the 11 preferred practices stratified by trial stage and funding. [file 1745-6215-12-9-S2.DOC]

**Additional file 2: Adherence to the 11 preferred practices stratified by trial stage and funding**

| **Practices** | **Trial Funding** | | | | | | | | | | | | | | | | | | | | |
| --- | --- | --- | --- | --- | --- | --- | --- | --- | --- | --- | --- | --- | --- | --- | --- | --- | --- | --- | --- | --- | --- |
|  |  | | **Both** | | | | | | | | | **Single** | | | | | | | | | |
|  |  |  |  |  |  |  |  |  |  |  |  |  |  |  |  |  |  |  |  |  |  |
|  | **Overall** | | **Non-industry** | | | **Industry** | | **P-Value ^*^** | | | | **Non-industry** | | | | **Industry** | | **P-Value ^†^** | | | |
|  | **(N=700)** | | **(N=406)** | | | **(N=406)** | |  | | | | **(N=240)** | | | | **(N=54)** | |  | | | |
|  | **n** | **(%)** | **n** | | **(%)** | **n** | **(%)** |  | | | | **n** | | | **(%)** | **n** | **(%)** |  | | | |
| ***Trial Preparation Stage*** | | | | | | | | | | | | | | | | | | | | | |
| **Signed contracts reviewed by institution ^‡^** |  | |  | | |  | |  | | | |  | | | |  | |  | | | |
| Signed contracts | 458 | | 187 | | | 337 | |  | | | | 75 | | | | 39 | |  | | | |
| No trials | 13 | (3) | 7 | | (4) | 6 | (2) |  | |  | | 3 | | | (4) | 4 | (10) |  |  | | |
| Some trials | 39 | (9) | 16 | | (9) | 17 | (5) |  | | 0.06 | | 5 | | | (7) | 1 | (3) |  | 0.36 | | |
| All trials ^§^ | 374 | (82) | 145 | | (78) | 298 | (88) |  | |  | | 46 | | | (61) | 32 | (82) |  |  | | |
| Not sure | 22 | (5) | 19 | | (10) | 12 | (4) |  | | | | 13 | | | (17) | 0 | (0) |  | | | |
| Did not answer | 10 | (2) | 0 | | (0) | 4 | (1) |  | | | | 8 | | | (11) | 2 | (5) |  | | | |
| **Signed contracts have restrictive confidentiality clauses ^‡^** |  |  |  | |  |  |  |  | | | |  | | |  |  |  |  | | | |
| Signed contracts | 458 | | 187 | | | 337 | |  | | | | 75 | | | | 39 | |  | | | |
| No trials ^§^ | 54 | (12) | 28 | | (15) | 24 | (7) |  |  | | | 20 | | | (27) | 4 | (10) |  | |  | |
| Some trials | 99 | (22) | 29 | | (16) | 51 | (15) |  | <0.001 | | | 6 | | | (8) | 7 | (18) |  | | 0.005 | |
| All trials | 201 | (44) | 64 | | (34) | 194 | (58) |  |  | | | 13 | | | (17) | 18 | (46) |  | |  | |
| Not sure | 94 | (21) | 66 | | (35) | 64 | (19) |  | | | | 28 | | | (37) | 8 | (21) |  | | | |
| Did not answer | 10 | (2) | 0 | | (0) | 4 | (1) |  | | | | 8 | | | (11) | 2 | (5) |  | | | |
| **Budgetary reviewed by a research ethics board or institution official** |  | |  | | | | |  | | | |  | | | |  | |  | | | |
| No trials | 39 | (6) | 27 | | (7) | 19 | (5) |  |  | | | 22 | | | (9) | 3 | (6) |  | |  | |
| Some trials | 92 | (13) | 40 | | (10) | 24 | (6) |  | 0.06 | | | 16 | | | (7) | 4 | (7) |  | | 0.65 | |
| All trials ^§^ | 523 | (75) | 323 | | (80) | 342 | (84) |  |  | | | 164 | | | (68) | 44 | (81) |  | |  | |
| Not sure | 28 | (4) | 16 | | (4) | 17 | (4) |  | | | | 22 | | | (9) | 1 | (2) |  | | | |
| Did not answer | 18 | (3) | 0 | | (0) | 4 | (1) |  | | | | 16 | | | (6) | 2 | (4) |  | | | |
| **Trials registered in trial registry since 2005** |  |  |  | |  |  |  |  | | | |  | | |  |  |  |  | | | |
| No trials | 50 | (7) | 37 | | (9) | 29 | (7) |  | |  | | 19 | | | (8) | 7 | (13) |  | |  | |
| Some trials | 221 | (32) | 104 | | (26) | 62 | (15) |  | | 0.18 | | 69 | | | (29) | 7 | (13) |  | | 0.09 | |
| All trials ^§^ | 274 | (39) | 147 | | (36) | 121 | (30) |  | |  | | 107 | | | (45) | 20 | (37) |  | |  | |
| Not sure | 140 | (20) | 102 | | (25) | 176 | (43) |  | | | | 36 | | | (15) | 17 | (31) |  | | | |
| Did not answer | 15 | (2) | 16 | | (4) | 18 | (4) |  | | | | 9 | | | (4) | 3 | (6) |  | | | |
| ***Trial Conduct Stage*** | | | | | | | | | | | | | | | | | | | | | |
| **Funder owns study data** |  |  |  | |  |  |  |  | | | |  | | |  |  |  |  | | | |
| No trials ^§^ | 258 | (37) | 232 | | (57) | 46 | (11) |  | |  | | 162 | | | (68) | 6 | (11) |  | |  | |
| Some trials | 221 | (32) | 33 | | (8) | 106 | (26) |  | | <0.001 | | 9 | | | (4) | 8 | (15) |  | | <0.001 | |
| All trials | 107 | (15) | 52 | | (13) | 148 | (36) |  | |  | | 16 | | | (7) | 24 | (44) |  | |  | |
| Not sure | 87 | (12) | 89 | | (22) | 102 | (25) |  | | | | 30 | | | (13) | 12 | (22) |  | | | |
| Did not answer | 27 | (4) | 0 | | (0) | 4 | (1) |  | | | | 23 | | | (9) | 4 | (7) |  | | | |
| **Investigator has access to data from all sites** |  |  |  | |  |  |  |  | | | |  | | |  |  |  |  | | | |
| No trials | 80 | (11) | 34 | | (8) | 54 | (13) |  |  | | | 35 | | | (15) | 7 | (13) |  |  | | |
| Some trials | 191 | (27) | 68 | | (17) | 95 | (23) |  | <0.001 | | | 26 | | | (11) | 13 | (24) |  | <0.001 | | |
| All trials ^§^ | 265 | (38) | 184 | | (45) | 92 | (23) |  |  | | | 122 | | | (51) | 7 | (13) |  |  | | |
| Not sure | 132 | (19) | 116 | | (29) | 159 | (39) |  | | | | 31 | | | (13) | 22 | (41) |  | | | |
| Did not answer | 32 | (5) | 4 | | (1) | 6 | (1) |  | | | | 26 | | | (11) | 5 | (9) |  | | | |
| **Funder controls final decisions regarding:** |  |  |  | |  |  |  |  | | | |  | | |  |  |  |  | | | |
| **Study design** |  |  |  | |  |  |  |  | | | |  | | |  |  |  |  | | | |
| No trials ^§^ | 247 | (35) | 216 | | (53) | 69 | (17) |  |  | | | 150 | | | (63) | 9 | (17) |  |  | | |
| Some trials | 228 | (33) | 41 | | (10) | 98 | (24) |  | <0.001 | | | 5 | | | (2) | 14 | (26) |  | <0.001 | | |
| All trials | 141 | (20) | 81 | | (20) | 158 | (39) |  |  | | | 37 | | | (15) | 21 | (39) |  |  | | |
| Not sure | 63 | (9) | 67 | | (17) | 77 | (19) |  | | | | 31 | | | (13) | 7 | (13) |  | | | |
| Did not answer | 21 | (3) | 1 | | (0) | 4 | (1) |  | | | | 17 | | | (7) | 3 | (6) |  | | | |
| **Data analysis** |  |  |  | |  |  |  |  | | | |  | | |  |  |  |  | | | |
| No trials ^§^ | 276 | (39) | 237 | | (58) | 82 | (20) |  |  | | | 160 | | | (67) | 10 | (19) |  |  | | |
| Some trials | 222 | (32) | 30 | | (7) | 100 | (25) |  | <0.001 | | | 7 | | | (3) | 14 | (26) |  | <0.001 | | |
| All trials | 120 | (17) | 76 | | (19) | 136 | (33) |  |  | | | 33 | | | (14) | 19 | (35) |  |  | | |
| Not sure | 61 | (9) | 62 | | (15) | 84 | (21) |  | | | | 23 | | | (10) | 8 | (15) |  | | | |
| Did not answer | 21 | (3) | 1 | | (0) | 4 | (1) |  | | | | 17 | | | (7) | 3 | (6) |  | | | |
| **Data interpretation** |  |  |  | |  |  |  |  | | | |  | | |  |  |  |  | | | |
| No trials ^§^ | 300 | (43) | 242 | | (60) | 92 | (23) |  |  | | | 162 | | | (68) | 11 | (20) |  |  | | |
| Some trials | 207 | (30) | 29 | | (7) | 97 | (24) |  | <0.001 | | | 7 | | | (3) | 14 | (26) |  | <0.001 | | |
| All trials | 106 | (15) | 76 | | (19) | 112 | (28) |  |  | | | 30 | | | (13) | 14 | (26) |  |  | | |
| Not sure | 66 | (9) | 58 | | (14) | 101 | (25) |  | | | | 24 | | | (10) | 12 | (22) |  | | | |
| Did not answer | 21 | (3) | 1 | | (0) | 4 | (1) |  | | | | 17 | | | (7) | 3 | (6) |  | | | |
| ***Trial Dissemination Stage*** | | | | | | | | | | | | | | | | | | | | | |
| **Funder controls final decision on content of submitted manuscripts** |  |  |  | |  |  |  |  | | | |  | | |  |  |  |  | | | |
| No trials ^§^ | 368 | (53) | 265 | | (65) | 108 | (27) |  |  | | | 180 | | | (75) | 16 | (30) |  |  | | |
| Some trials | 168 | (24) | 30 | | (7) | 92 | (23) |  | <0.001 | | | 7 | | | (3) | 8 | (15) |  | <0.001 | | |
| All trials | 49 | (7) | 30 | | (7) | 65 | (16) |  |  | | | 11 | | | (5) | 5 | (9) |  |  | | |
| Not sure | 88 | (13) | 81 | | (20) | 136 | (34) |  | | | | 19 | | | (8) | 21 | (39) |  | | | |
| Did not answer | 27 | (4) | 0 | | (0) | 5 | (1) |  | | | | 23 | | | (10) | 4 | (7) |  | | | |
| **Completed manuscripts has ghost authorship** |  |  |  | |  |  |  |  | | | |  | | |  |  |  |  | | | |
| No trials ^§^ | 450 | (64) | 295 | | (73) | 126 | (31) |  |  | | | 183 | | | (76) | 21 | (39) |  |  | | |
| Some trials | 100 | (14) | 26 | | (6) | 70 | (17) |  | <0.001 | | | 9 | | | (4) | 5 | (9) |  | 0.029 | | |
| All trials | 5 | (1) | 1 | | (0) | 8 | (2) |  |  | | | 3 | | | (1) | 0 | (0) |  |  | | |
| Not sure | 117 | (17) | 82 | | (20) | 196 | (48) |  | | | | 22 | | | (9) | 24 | (44) |  | | | |
| Did not answer | 28 | (4) | 2 | | (0) | 6 | (1) |  | | | | 23 | | | (10) | 4 | (7) |  | | | |

Notes:

^*^ Analyzed using repeated measures logistic regression model.

^†^ Analyzed using Fisher’s exact test

^‡^ Question was related to 458 investigators who had signed contracts.

^§^ Rows indicated the proportion of investigators that reported full adherence to good trial practices for all of their trials
